# Genomic Analysis of Two Phylogenetically Distinct *Nitrospira* Species Reveals Their Genomic Plasticity and Functional Diversity

**DOI:** 10.3389/fmicb.2017.02637

**Published:** 2018-01-09

**Authors:** Norisuke Ushiki, Hirotsugu Fujitani, Yu Shimada, Tomohiro Morohoshi, Yuji Sekiguchi, Satoshi Tsuneda

**Affiliations:** ^1^Department of Life Science and Medical Bioscience, Waseda University, Tokyo, Japan; ^2^Department of Material and Environmental Chemistry, Graduate School of Engineering, Utsunomiya University, Tochigi, Japan; ^3^Bio-Measurement Research Group, Biomedical Research Institute, National Institute of Advanced Industrial Science and Technology, Ibaraki, Japan

**Keywords:** *Nitrospira*, nitrification, genome, autoinducer, activated sludge

## Abstract

The genus *Nitrospira* represents a dominant group of nitrite-oxidizing bacteria in natural and engineered ecosystems. This genus is phylogenetically divided into six lineages, for which vast phylogenetic and functional diversity has been revealed by recent molecular ecophysiological analyses. However, the genetic basis underlying these phenotypic differences remains largely unknown because of the lack of genome sequences representing their diversity. To gain a more comprehensive understanding of *Nitrospira*, we performed genomic comparisons between two *Nitrospira* strains (ND1 and NJ1 belonging to lineages I and II, respectively) previously isolated from activated sludge. In addition, the genomes of these strains were systematically compared with previously reported six *Nitrospira* genomes to reveal their similarity and presence/absence of several functional genes/operons. Comparisons of *Nitrospira* genomes indicated that their genomic diversity reflects phenotypic differences and versatile nitrogen metabolisms. Although most genes involved in key metabolic pathways were conserved between strains ND1 and NJ1, assimilatory nitrite reduction pathways of the two *Nitrospira* strains were different. In addition, the genomes of both strains contain a phylogenetically different urease locus and we confirmed their ureolytic activity. During gene annotation of strain NJ1, we found a gene cluster encoding a quorum-sensing system. From the enriched supernatant of strain NJ1, we successfully identified seven types of acyl-homoserine lactones with a range of C10–C14. In addition, the genome of strain NJ1 lacks genes relevant to flagella and the clustered regularly interspaced short palindromic repeat (CRISPR)-Cas (CRISPR-associated genes) systems, whereas most nitrifying bacteria including strain ND1 possess these genomic elements. These findings enhance our understanding of genomic plasticity and functional diversity among members of the genus *Nitrospira*.

## Introduction

Nitrification is a key aerobic process of the nitrogen cycle, which is catalyzed by chemolithoautotrophic ammonia-oxidizing archaea, ammonia-oxidizing bacteria, and nitrite-oxidizing bacteria (NOB). NOB converting nitrite to nitrate prevent toxic nitrite accumulation (Philips et al., 2002) and produce nitrate, which is an important source of nitrogen assimilation by many microorganisms and plants. Moreover, NOB contribute to the biological nitrogen removal process of wastewater treatment plants (WWTPs), preventing costal ecosystem eutrophication (Okabe et al., 2011).

To date, it has been reported that the chemolithoautotrophic NOB comprise seven genera (*Nitrobacter, Nitrococcus, Nitrospina, Nitrospira, Nitrotoga, Nitrolancea*, and *Candidatus* Nitromaritima) (Daims et al., 2016). Of these NOB, the genus *Nitrospira* is globally distributed in soils, oceans, freshwater habitats, hot springs, and WWTPs (Anitori et al., 2002; Altmann et al., 2003; Freitag et al., 2005; Lebedeva et al., 2005; Hongxiang et al., 2008; Neilson et al., 2012; Pester et al., 2014; Gruber-Dorninger et al., 2015). The comparative analysis of 16S rRNA gene sequences revealed that the genus *Nitrospira* was classified into six different phylogenetic lineages (Daims et al., 2001; Lebedeva et al., 2011), which reflected the diversity of *Nitrospira* habitats (Daims et al., 2006). Remarkably, *Nitrospira* lineages I and II were ubiquitously detected as dominant NOB in the activated sludge of WWTPs with competition and the partitioning of ecological niches between phylogenetically different populations (Daims et al., 2001; Maixner et al., 2006; Gruber-Dorninger et al., 2015; Lücker et al., 2015). However, *Nitrospira* is notoriously recalcitrant to isolation and representative *Nitrospira* strains from activated sludge were limited. Recently, the development of a separation technique using optical tweezers or a cell sorting system enabled the isolation of *Nitrospira defluvii, Nitrospira lenta, Nitrospira* sp. ND1, and *Nitrospira japonica* from WWTPs (Ushiki et al., 2013; Fujitani et al., 2014; Nowka et al., 2015b), and their physiological properties and kinetic parameters were partially investigated (Nowka et al., 2015a; Ushiki et al., 2017).

Recently, the first metagenomic analysis of *N. defluvii* belonging to lineage I revealed a novel nitrite-oxidoreductase, which is a key enzyme involved in nitrite oxidation by *Nitrospira*, and predicted that *Nitrospira* evolved from microaerophilic or anaerobic ancestors (Lücker et al., 2010). Subsequently, genomic analysis and physiological characterization of *Nitrospira moscoviensis* belonging to lineage II revealed growth of this organism by aerobic hydrogen oxidation (Koch et al., 2014) or the aerobic use of formate (Koch et al., 2015) without nitrite oxidation. Moreover, the wide distribution of environmental ureases and cyanases among *Nitrospira* was discovered by genomic analysis, suggesting that NOB could supply ammonia oxidizers lacking ureases and cyanases with ammonia and carbonate (Koch et al., 2015; Palatinszky et al., 2015). Surprisingly, it was reported that some *Nitrospira* species belonging to lineage II were able to perform complete ammonia oxidation (COMAMMOX), which oxidized ammonia to nitrate by a single microorganism (Daims et al., 2015; van Kessel et al., 2015). In addition, four COMAMMOX and one canonical *Nitrospira* were recovered from metagenomes, and the comparative genomic analysis of COMAMMOX and related nitrifiers revealed their niche differentiation and the evolutionary history (Palomo et al., unpublished). These genomic analyses of *Nitrospira* have revealed their metabolic versatility, indicating that the ecophysiological role of *Nitrospira* is not exclusively nitrite oxidation.

As mentioned above, genomic analyses are powerful molecular approaches for finding unexpected metabolic versatility and predicting the evolution of unknown microorganisms. Although the complete genome sequences of representative *Nitrospira* strains belonging to lineages I and II, and several COMAMMOX *Nitrospira* genomes had previously been reported (Lücker et al., 2010; Daims et al., 2015; Koch et al., 2015; Pinto et al., 2015; van Kessel et al., 2015; Chao et al., 2016; Camejo et al., 2017), available information of *Nitrospira* genome sequences is still insufficient to understand their metabolism and evolution. In this study, we conducted genomic analysis of two *Nitrospira* strains, *Nitrospira* sp. strain ND1 and *N. japonica* strain NJ1, belonging to lineages I and II, respectively, both of which were previously isolated from activated sludge from a WWTP by our research group (Ushiki et al., 2013; Fujitani et al., 2014), and compared their genomic information with those of previously reported *Nitrospira* strains. Our findings revealed remarkable heterogeneity in the genes involved in quorum-sensing systems, bacterial flagella and CRISPR-Cas system, and indicated that the phylogenetic diversity of *Nitrospira* correlated to adaptation to various environments.

## Materials and Methods

### Genome Reconstruction and Annotation

*Nitrospira* sp. strain ND1 and *N. japonica* strain NJ1 were isolated from activated sludge from a WWTP as previously reported (Ushiki et al., 2013; Fujitani et al., 2014). DNA was extracted from strain ND1 or strain NJ1 using a NucleoSpin^®^ Tissue DNA extraction kit (Takara Bio, Otsu, Japan) according to the manufacturer’s instructions. DNA sequencing for generating the genomes of the strains was performed by National Institute of Advanced Science and Technology. Briefly, paired-end (300 to 1,000 bp inserts, Nextera XT indexed) and Nextera mate-pair (1 to 14 kbp insert) libraries were generated and sequenced on an Illumina MiSeq instrument using V2 chemistry (2-bp × 250-bp reads). Raw reads were merged with SeqPrep using default settings, including the removal of sequencing adapters. Reads that failed to merge were quality trimmed and filtered using Nesoni v0.112. Both merged and processed single reads were assembled using SPAdes version 2.5.0 (Bankevich et al., 2012), followed by manual curation of the assembly (Sekiguchi et al., 2015). The reconstructed genome sequences of strains ND1 and NJ1 were deposited in the European Nucleotide Archive (ENA) under the accession numbers FWEX01000001-FWEX01000006 and LT828648 respectively.

The reconstructed draft and complete genomes of the strains ND1 and NJ1, respectively, were integrated into the MicroScope platform (Vallenet et al., 2013), and associated annotations are publicly available in MicroScope (#17KHXP and #3D7KKV respectively). Coding sequences (CDS) were automatically predicted and annotated by using the MicroScope platform. In addition, function of the annotated CDS as hypothetical protein by the MicroScope platform was speculated by searching their similar proteins with BLAST in NCBI Reference proteins database. The predicted CDS were summarized into a table according to their predicted function, which included nitrogen metabolism, carbon fixation, respiration, and motility (Supplementary Data Sheet S2). CRISPR repeat sequences were identified using CRISPRfinder (Grissa et al., 2007).

### Comparison of *Nitrospira* Genome Sequences

**FIGURE 1:**
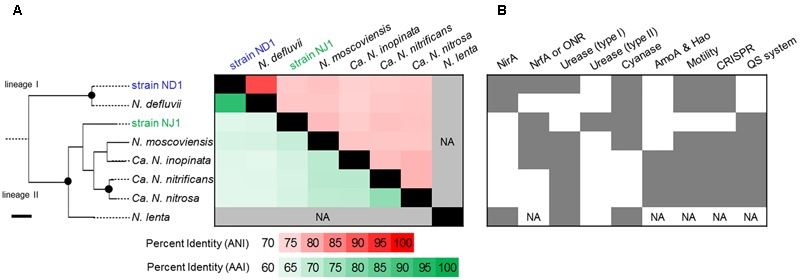
Comparison of the average nucleotide identity (ANI), average amino acid identity (AAI), and presence/absence of several functional genes/operons among the several *Nitrospira* genomes. The phylogenetic tree based on 16S rRNA gene sequences of *Nitrospira* strains was constructed using the Maximum Likelihood algorithm. Black dots indicate high (>90%) parsimony bootstrap values (500 interactions) supporting each clade. The scale bar corresponds to 1% estimated nucleotide sequence divergence. **(A)** Heatmap showing the ANI (upper diagonal) and the AAI (lower diagonal). The heatmap was ordered phylogenetically based on similarities between the 16S rRNA sequences. **(B)** Presence of several functional genes/operons is shown in dark gray. NirA, ferredoxin-nitrite reductase; NrfA or ONR, cytochrome *c* nitrite reductase; Urease (type I), urease operon; Urease (type II), phylogenetically different urease operon to type I; Cyanase, cyanate hydratase; Amo and Hao; ammonia monooxygenase and hydroxylamine oxidase; Motility, potential using bacterial flagella; CRISPR, CRISPR-Cas systems; QS system, autoinducer synthase. NA indicates data that was not available.

The average nucleotide identity (ANI) and the average amino acid identity (AAI) among *Nitrospira* genome sequences were calculated using online tools (Rodriguez-R and Konstantinidis, 2016). The parameters of the ANI calculation were adjusted to the following default settings: a minimum alignment length, a minimum identity, a fragment window size, and a fragment step size of 700 bp, 70%, 1,000 bp, and 200 bp, respectively. The minimum identity of the AAI calculation was adjusted to 30%. Homologous proteins between two *Nitrospira* strains were identified by using the phyloprofile exploration tool of MicroScope (Vallenet et al., 2013). The parameters of the phyloprofile exploration were adjusted to the following default settings: a minimum alignment length and a minimum identity of 80 and 30%, respectively. In addition, based on gene annotation information of the *Nitrospira* genomes, the presence/absence of several functional genes/operons among the genomes was summarized into a figure (**Figure 1**).

### Phylogenetic Analysis

Reference 16S rRNA and protein sequences for phylogenetic inference were obtained from the NCBI database. Multiple sequence alignments of these sequences were generated automatically using ClustalW2 (Larkin et al., 2007). Phylogenetic trees based on 16S rRNA gene sequences were constructed using MEGA 6 software with the Maximum Likelihood algorithm (Tamura et al., 2013). Also, phylogenetic trees were constructed for the amino acid sequences of selected proteins using the Maximum Likelihood algorithm.

### Identification of AHLs in *Nitrospira japonica* Strain NJ1

Preparation for identification of AHLs using the LC–MS/MS was conducted according to previous studies (Burton et al., 2005; Mellbye et al., 2016). First, the 1 L batch cultures of strain NJ1 were acidified to pH < 2 with HCl to avoid lactonolysis. Then the acidified samples were extracted five times with 200 ml of ethyl acetate. The extracted samples were dried down using an evaporator and recovered with 1 mL dimethylsulfoxide. Then, according to the previous study (Okutsu et al., 2016; Morohoshi et al., 2016), identification of the extracted AHLs was performed using a triple quadrupole/linear ion trap instrument (QTRAP5500, AB Sciex, Framingham, MA, United States) with an electrospray ionization source coupled to an UHPLC system (Nexera X2, Shimadzu, Kyoto, Japan). Chromatographic separation was achieved on a C18 column (Kinetex F5, Φ 2.1 mm × 150 mm, 2.6 μm, Phenomenex, Torrance, CA, United States). MS/MS spectra were recorded as reported previously (Morohoshi et al., 2016). Precursor ion scanning experiments were performed in positive ion mode for analysis. In the experiments, one quadrupole (Q1) was set to scan a mass range of m/z 80 to 500 Da and other quadrupole (Q3) was used for detection of the product ion at m/z 102, which indicates presence of lactone ring in the compound (Okutsu et al., 2016). AHLs were identified by precursor ion scanning experiments and comparison with synthetic AHL standards. AHL standards, C10-HSL, 3-oxo-C10-HSL, C12-HSL, 3-oxo-C12-HSL, 3-OH-C12-HSL, C14-HSL, and 3-oxo-C14-HSL, were synthesized using a previously described method (Morohoshi et al., 2013). Purity of these AHL standards was checked by using nuclear magnetic resonance (NMR).

## Results and Discussion

### Genomic Comparison of *Nitrospira* Cultures

The final high-quality draft genome assembly of the ND1 genome consisted of six contigs. The largest and N50 contig size was 3,463,908 bp, and the total assembly size was 4,454,928 bp for the ND1 genome. The obtained genome sequence of strain ND1 contained 4,612 CDS (Supplementary Table S1). By contrast, the obtained sequence reads from strain NJ1 were assembled into one scaffold. The reconstructed complete genome sequence of strain NJ1 was 4,084,817 bp in length and contained 4,150 predicted CDS (Supplementary Table S1). These two *Nitrospira* strains appeared to contain no plasmids.

We compared the two reconstructed genome sequences with the published complete genome sequences of *N. defluvii* (NC_014355), *N. moscoviensis* (NZ_CP011801), and *Nitrospira inopinata* (NZ_LN885086), and published contig sequences of *Candidatus* Nitrospira nitrosa (CZQA01000001_CZQA01000015) and *Candidatus* Nitrospira nitrificans (CZPZ01000001_CZPZ01000036), based on 16S rRNA gene similarity, ANI, AAI, and the presence/absence of several functional genes. Phylogenetic analysis based on the 16S rRNA gene revealed that strain ND1 and the *N. defluvii* strain belonged to lineage I, and the other five *Nitrospira* strains belonged to lineage II (**Figure 1**). Strain ND1 shares a similar 16S rRNA gene sequence to *N. defluvii* (DQ059545; 99.8%) (Spieck et al., 2006). Strain NJ1 is distantly related to *N. moscoviensis* (CP011801; 96.1%) (Ehrich et al., 1995) and *N. lenta* (KF724505; 96.1%) (Nowka et al., 2015b), and is also distantly related to strain ND1 (92.6%). The ANI and AAI among *Nitrospira* genome sequences indicated a close genetic relatedness between strain ND1 and *N*. *defluvii* (91.4%, **Figure 1A**). But, the genome sequence of strain ND1 contains genes encoding cytochrome *c* nitrite reductase (NrfA) and a urease locus, neither of which is contained in the genome of *N. defluvii* (**Figure 1B**). By contrast, the ANI and AAI among the genome sequence of *Nitrospira* strains belonging to lineage II were below 80%, suggesting a putative functional diversity due to the evolutionary distance of the different *Nitrospira* lineage II genomes (**Figure 1A**). Also, the strain ND1 genome contained 2,717 out of 4,612 CDS as homologous genes shared by the strain NJ1 genome, suggesting that their genomes contained a considerably different set of genes despite being isolated from the same activated sludge (**Figure 1A**).

**FIGURE 2:**
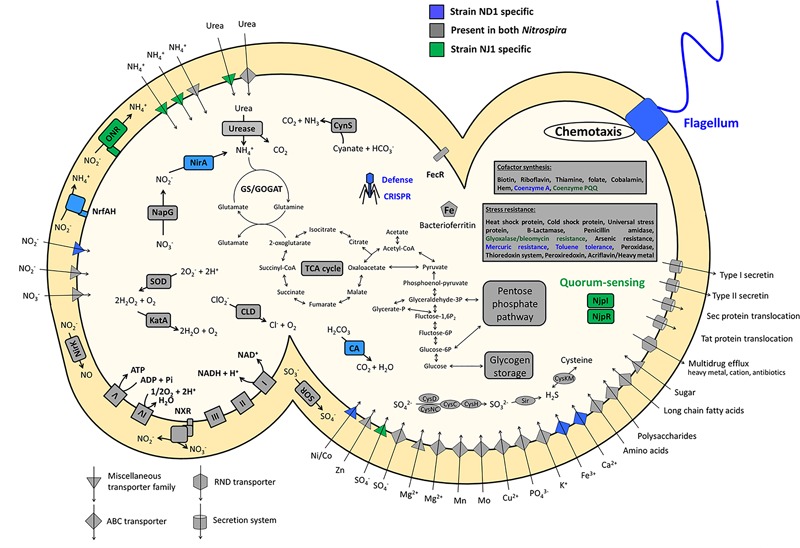
Schematic representation of a cell showing the key and unique metabolic features encoded by the genomes of strains ND1 and NJ1. Core functions shared between the two *Nitrospira* isolates are shown in gray, specific features of strain ND1 are shown in blue, and those of strain NJ1 are shown in green. CA, carbonic anhydrase; CLD, chlorite dismutase; CRISPR, clustered regularly interspaced short palindromic repeats; CynS, cyanase; GS/GOGAT, glutamine synthetase/glutamate synthase (glutamine oxoglutarate aminotransferase); KatA, catalase; NapG, nitrate reductase; NirA, ferredoxin-nitrite reductase; NirK, copper-containing nitrite reductase (forming NO); NjpI, autoinducer synthase; NjpR, autoinducer receptor; NrfAH, cytochrome *c* nitrite reductase; ONR, octaheme cytochrome *c* nitrite reductase; SOR, cytochrome *c* sulfite oxidoreductase; SOD, superoxide dismutase. Enzyme complexes of the electron transport chain are labeled by Roman numerals. The TCA cycle depicts both directions.

The CDS annotated with unknown function of the genome sequence in the strains ND1 and NJ1 accounted for 40.5 and 41.5%, respectively. Based on the annotated CDS with certain function in the genome sequence of the two strains, their key metabolic pathways were predicted and compared with other *Nitrospira* strains. Comparing genomic features among *Nitrospira* strains, we first focused on the genes involved in nitrogen metabolism, such as nitrite reduction and urea degradation. Next, from the strain NJ1 genome, we discovered the key genes of acyl-homoserine lactone (AHL)-type quorum-sensing (QS) systems, which was reported in the previous study (Nasuno et al., 2012; Mellbye et al., 2017), and we identified the chemical structures of the AHLs as autoinducers of strain NJ1. Moreover, the genome sequence of strain NJ1 lacks genes encoding bacterial flagella and CRISPR-Cas system, which were reported in genomic analyses of other *Nitrospira* (**Figure 2**).

### Nitrogen Metabolism of Strains ND1 and NJ1

*Nitrospira* is a chemolithoautotrophic nitrite-oxidizing bacterium, and it was confirmed that strains ND1 and NJ1 were able to grow in mineral medium with oxidizing nitrite to nitrate in the previous reports (Ushiki et al., 2013; Fujitani et al., 2014). Both strains appeared to oxidize nitrite for energy conservation and to assimilate nitrite as a nitrogen source. Although most genes involved in key metabolic pathways (nitrite oxidation, the electron transport chain, glycolysis/gluconeogenesis, the tricarboxylic acid cycle, the pentose phosphate pathway, and sulfur assimilation) were conserved between the two *Nitrospira* genome sequences, genes involved in nitrogen assimilation differed (**Figure 2** and Supplementary Data Sheet S2). The genome sequence of strain NJ1 contained genes encoding an Octaheme cytochrome *c* nitrite reductase (ONR) and a cytochrome *bc* complex (**Figure 2**). Previously, it was reported that nitrite reduction in *N. moscoviensis* was most likely catalyzed by ONR (Koch et al., 2015). Meanwhile, the genome sequence of strain ND1 contained two nitrite reductase genes, *nirA* and *nrfAH* (**Figure 2**). NirA is reported to be a cytoplasmic ferredoxin-dependent nitrite reductase for nitrogen assimilation in plant cells and prokaryote cells (Luque et al., 1993; Suzuki et al., 1993; Moreno-Vivian et al., 1999). By contrast, NrfAH is a periplasmic cytochrome *c* nitrite reductase using electrons from quinol, not for assimilation, but rather for respiratory ammonification (Simon, 2002). Previous studies reported that nitrite reduction in *N. defluvii* and *N. lenta* was most likely catalyzed by NirA (Lücker et al., 2010; Koch et al., 2015), whereas Daims et al. (2015) reported the presence of NrfAH in the genome of *N. inopinata*, but nitrite reduction by NrfAH was not shown.

**FIGURE 3:**
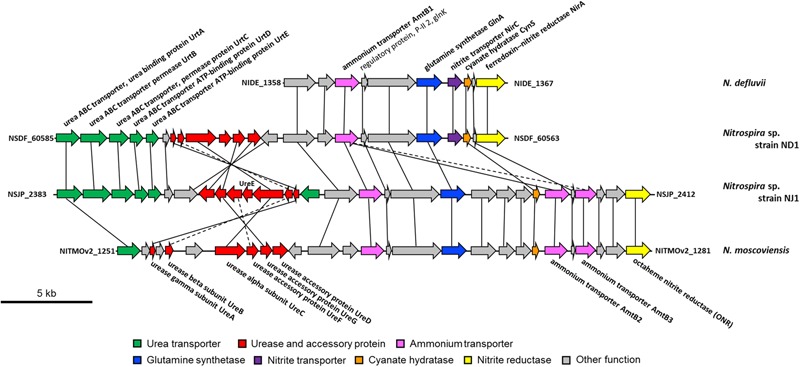
Schematic representation of the genomic regions in *Nitrospira defluvii*, strain ND1, strain NJ1, and *Nitrospira moscoviensis* containing the genes encoding the urea transporter, urease, urea accessory protein, ammonium transporter, glutamine synthetase, nitrite transporter, cyanate hydratase, and nitrite reductase. Solid lines connect homologous genes that encode proteins sharing sequence similarities above 50%. Dashed lines connect genes that encode proteins sharing sequence similarities between 30 and 50%.

Comparing the genomic region of genes involved in nitrogen metabolism among *Nitrospira* strains, the gene encoding ONR in strain NJ1 and *N. moscoviensis*, and the *nirA* gene in strain ND1 and *N. defluvii* are located close to the *glnA* gene encoding glutamine synthetase, the *amtB* genes encoding an ammonium transporter, and the *glnK* gene encoding P-II protein, which are known to be involved in nitrogen assimilation (**Figure 3**). Thus, nitrite reduction in strains ND1 and NJ1 was most likely catalyzed by NirA and ONR, respectively. Indeed, expression of the gene encoding ONR of strain NJ1 was confirmed by reverse-transcription (RT)-PCR (Supplementary Figure S1 and Supplementary Table S2). By contrast, the *nrfAH* gene (NSND_62628, NSND_62627, NITINOP_0678, and NITINOP_0679) in the genome sequence of strain ND1 and *N. inopinata* was not located close to the *glnA* gene (NSND_60567 and NITINOP_1778). Likely, their NrfAH was not used for assimilatory nitrite reduction. The genome sequence of strain ND1 and *N. inopinata* contain *fdhABC* gene (NSND_50216-NSND_50218) encoding formate dehydrogenase and *hydABC* gene (NITINOP_0586-NITINOP_0588) encoding hydrogenase respectively, which are able to use formate and hydrogen as electron donor of respiratory ammonification (Simon, 2002). Besides, it was reported that *Nitrospira* strains grow with formate or hydrogen as substrate (Ushiki et al., 2013; Koch et al., 2014, 2015). Although we did not check their respiratory ammonification with formate and hydrogen, the NrfAH shared among *Nitrospira* genomes might be required for respiratory ammonification.

**FIGURE 4:**
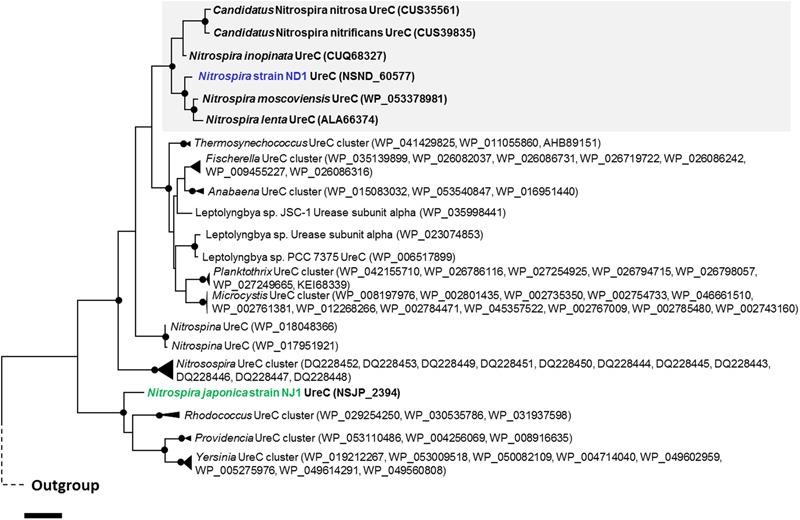
Phylogenetic affiliation of urease alpha subunit from *Nitrospira* and other nitrifiers. The phylogenetic tree was constructed using the Maximum Likelihood algorithm. Black dots indicate high (>90%) parsimony bootstrap values (500 interactions) supporting each clade. The scale bar corresponds to 10% estimated amino acid sequence divergence.

Recently, it was reported that some *Nitrospira* strains performed not only nitrite oxidation, but also urea degradation, cyanate degradation, and ammonia oxidation (Daims et al., 2015; Koch et al., 2015; Palatinszky et al., 2015; van Kessel et al., 2015). Our reconstructed genome sequences of strains ND1 and NJ1 also contained genes involved in the degradation of urea and cyanate (**Figure 2**), and we confirmed ureolytic activity of both strains (Supplementary Figure S2). Phylogenetic analysis based on the urease alpha subunits (UreC) revealed that the UreC proteins of most *Nitrospira* species, except strain NJ1, were affiliated with one *Nitrospira* clade (**Figure 4**). In addition, although previously reported *Nitrospira* genomes and strain ND1 lack the *ureE* gene encoding a urease accessory protein, the strain NJ1 genome sequence contained a complete gene cluster encoding urease accessory proteins including the UreE protein (**Figure 3**). Besides, the genomes of both strains contained the high affinity urea ABC transporter (*urtABCDE*) shared among most *Nitrospira* genomes possessing a urease locus (Daims et al., 2015; Koch et al., 2015; Palatinszky et al., 2015; van Kessel et al., 2015; Palomo et al., unpublished), and the strain NJ1 genome contained the Yut-like urea transporter, which is a single component channel for facilitated diffusion of urea (Sebbane et al., 2002) and was not contained in other reported *Nitrospira* genomes.

Therefore, the phylogeny of UreC proteins and the presence of the *ureE* gene and a different urea transporter indicated that the ureolytic machinery of strain NJ1 differs from that of other *Nitrospira* species. Besides, in the phylogenetic tree based on UreC, the *Nitrospira* clade included not only the UreC of *N. moscoviensis, N. lenta, N. inopinata, Ca*. Nitrospira nitrosa, and *Ca*. Nitrospira nitrificans belonging to *Nitrospira* lineage II, but also the UreC of strain ND1 belonging to *Nitrospira* lineage I. Although the wide distribution of environmental ureases among *Nitrospira* was reported in the previous study (Koch et al., 2015), the reported and published *Nitrospira* genomes possessing a urease belong to only lineage II. So, the possession of the *ureC* gene in the genome of strain ND1 belonging to *Nitrospira* lineage I indicated that urease genes widely distribute among *Nitrospira*, not only lineage II.

### The Quorum-Sensing System of *N. japonica* Strain NJ1

**FIGURE 5:**
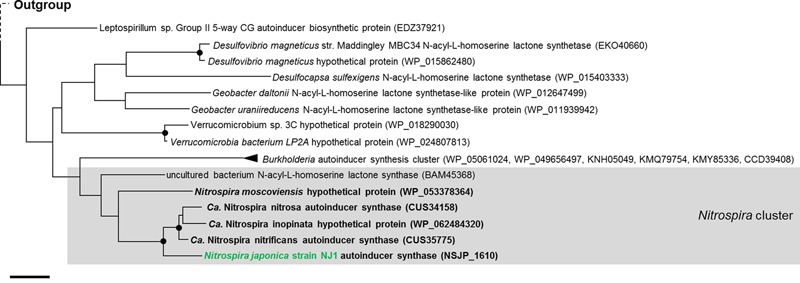
Phylogenetic affiliation of autoinducer synthase from strain NJ1 and other nitrifiers. The tree was constructed using the Maximum Likelihood algorithm. Black dots indicate high (>90%) parsimony bootstrap values (500 interactions) supporting each clade. The scale bar corresponds to 20% estimated amino acid sequence divergence.

Unexpectedly, the genome sequence of strain NJ1 contained the *njpI* gene (NSJP_1610) and the *njpR* gene (NSJP_1611) encoding a putative synthase and a receptor of autoinducers, respectively, which are known signaling compounds of QS systems (**Figure 2** and Supplementary Data Sheet S2). The QS system is a cell–cell communication mechanism among bacteria in environments that employs autoinducers to regulate bacterial behaviors such as biofilm formation, bacterial motility, luminescence, and plasmid transfer (Waters and Bassler, 2005; Hense and Schuster, 2015). AHLs, common signaling compounds of QS systems, are synthesized by the LuxI protein as autoinducer synthases. Previously, genes encoding a LuxI homolog and AHLs were identified in the genome sequences of *Nitrosomonas europaea* and *Nitrosospira multiformis*, which are known ammonia-oxidizing bacteria, and *Nitrobacter winogradskyi*, which is known as NOB (Burton et al., 2005; Gao et al., 2014; Mellbye et al., 2015). In addition, a metagenomic clone from the phylum *Nitrospirae* was reported to possess AubI/AubR as a LuxI/LuxR homolog, producing *N*-dodecanoyl-L-homoserinelactone (C12-HSL) as an autoinducer (Nasuno et al., 2012). Recently, it was reported that the genome sequence of *N. moscoviensis, N. inopinata*, and *Ca*. Nitrospira nitrificans contains a gene encoding AHL synthase homologs (Mellbye et al., 2017). Phylogenetic analysis based on the amino acid sequence of autoinducer synthases revealed that the LuxI homologs of *Nitrospira* were each clustered into single clades (**Figure 5**), and that NjpI, a LuxI homolog in strain NJ1, was most closely related to that of *Ca.* Nitrospira nitrificans (67.5%, CUS35775). But, since no gene encoding a LuxI homolog was detected in the genome sequence of strain ND1 or *N. defluvii* belonging to lineage I, the LuxI homologs might be partially distributed among the genomes of *Nitrospira* species.

### Identification of AHLs in Strain NJ1

**FIGURE 6:**
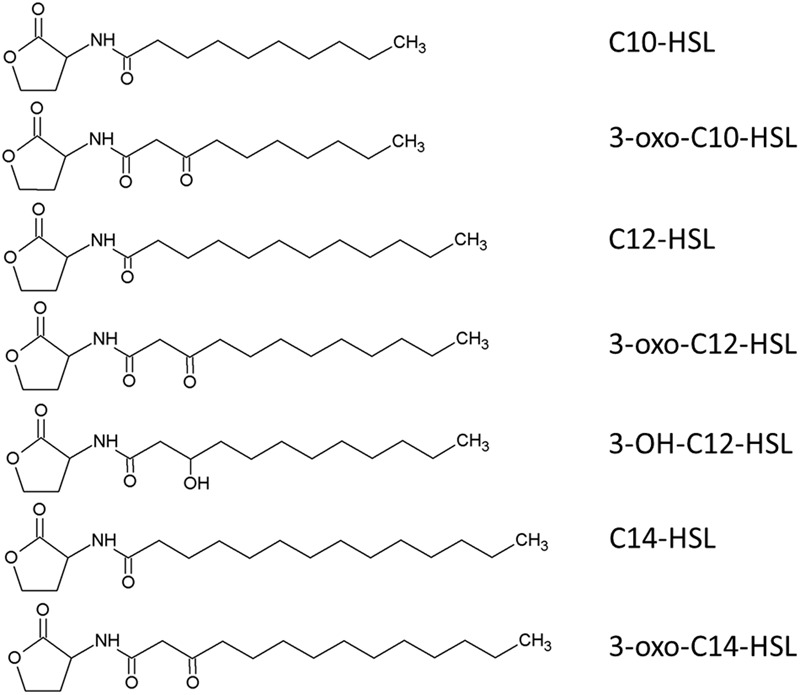
Predicted chemical structure of the seven AHLs obtained from the enriched supernatant of *Nitrospira japonica* strain NJ1.

We investigated AHL production in the culture supernatant of strain NJ1 using an AHL bioassay with *Chromobacterium violaceum* strain VIR07 (Morohoshi et al., 2008). We extracted AHLs from the batch culture of strain NJ1 according to the reported protocol (Burton et al., 2005). Using the AHL bioassay, AHLs were successfully detected from the enriched supernatant (Supplementary Figure S3). Subsequently, we identified the AHLs in the enriched supernatant using an LC–MS/MS. Unexpectedly, LC–MS/MS analysis revealed that seven types of AHLs, namely *N*-decanoyl-L-homoserine lactone (C10-HSL), *N*-(3-oxodecanoyl)-L-homoserine lactone (3-oxo-C10-HSL), *N*-dodecanoyl-L-homoserine lactone (C12-HSL), *N*-(3-hydroxydodecanoyl)-L-homoserine lactone (3-OH-C12-HSL), *N*-(3-oxododecanoyl)-L-homoserine lactone (3-oxo-C12-HSL), *N*-tetradecanoyl-L-homoserine lactone (C14-HSL), and *N*-(3-oxotetradecanoyl)-L-homoserine lactone (3-oxo-C14-HSL) were present in the supernatant (**Figure 6** and Supplementary Data Sheet S3). Comparison among relative abundance of these AHLs based on peak area of each compounds suggested that C12-HSL was the dominant autoinducer (Supplementary Table S3). Interestingly, although the genome sequence of strain NJ1 contains one *njpI* gene encoding an AHL synthase, the supernatant contained seven types of AHL. Previously, Shen et al. (2016) reported that several kinds of AHLs were identified from recombinant *Escherichia coli* cells containing the *nwiI* gene encoding an AHL synthase of *N. winogradskyi*. In addition, it was often reported that several types of AHLs were biosynthesized by only one AHL synthase from some bacteria, e.g., *Pseudomonas putida* WCS358 (Bertani and Venturi, 2004). It was also accepted that AHL synthase preferred to use not only one specific acyl carrier protein (ACP) charged with fatty acids, but also closely related acyl-ACPs, when AHLs were synthesized from *S*-adenosylmethionine and acyl-ACP (Cooley et al., 2008). Thus, the AHL synthase of strain NJ1 likely preferred to synthesize AHLs within a range of C10–C14. Comparing the chemical structures of the identified AHLs of strain NJ1 with those of nitrifying bacteria in previous studies (Supplementary Table S4), strain NJ1 appeared to synthesize and secrete different types of AHLs from other nitrifying bacteria. In addition, its dominant autoinducer, 12-HSL, was similar to that of a metagenomic clone from the phylum *Nitrospirae* (Nasuno et al., 2012). Thus, it was likely that partial *Nitrospira* strains might produce autoinducers conserved among *Nitrospira* species.

In recent studies, it was suggested that nitrogen metabolism and the flux of nitrogen oxide in *N. winogradskyi* were regulated by AHL signaling (Mellbye et al., 2016; Shen et al., 2016). Although the role of AHL-based QS system in strain NJ1 is still unknown, future transcriptional analyses using nitrifying pure cultures possessing QS systems may help to explain the mechanisms of transcriptional regulation and interspecies communication mediated by AHLs.

### Bacterial Flagella and Chemotaxis of Strain ND1

The genome sequence of strain ND1 contained almost all of the known flagella genes such as *flg, flh, fli*, and *motAB*, and the principal chemotaxis genes such as *che* and *MCP* (Supplementary Data Sheet S2). However, the strain ND1 genome curiously lacked the *flhDC* gene as a master transcriptional regulator of flagellation and chemotaxis (Kutsukake, 1997). Likewise, all *Nitrospira* genome published on the NCBI database also lacks the *flhDC* gene. Following genome analysis of *N. winogradskyi*, a canonical NOB, it was reported that the *flhDC* gene was lacking, and flagellation and chemotaxis in *N. winogradskyi* appeared to be differently regulated by other genes (Starkenburg et al., 2006). Interestingly, the genome of strain ND1 and *N. defluvii* contained a homologous gene encoding the transcription factor FleQ and the two-component system FleSR similar to *Pseudomonas aeruginosa* (Ritchings et al., 1995; Arora et al., 1997). It was also reported that FleQ activates the transcription of genes involved in flagellar biogenesis and the FleSR two-component system depending on σ54 (Dasgupta et al., 2003). Thus, flagellation and chemotaxis of strain ND1 and *N. defluvii* appeared to be controlled by FleQ as a master transcriptional regulator.

### Lack of Flagella in *N. japonica* Strain NJ1

Only two genes involved in bacterial flagella were encoded in the *N. japonica* strain NJ1 genome sequence, suggesting that strain NJ1 lacks the potential for motility using flagella (**Figure 2**). As mentioned above, the genome sequence of strain ND1 contained almost all of the genes involved in bacterial flagella. Likewise, genes involved in flagella were reported in genomic analyses of other *Nitrospira* (Lücker et al., 2010; Koch et al., 2015), and flagella of *N. inopinata* were recently observed by transmission electron microscopy (Daims et al., 2015). Moreover, genes encoding bacterial flagella have been reported in the genome sequences of the genera *Nitrobacter, Nitrococcus, Nitrospina, Nitrosomonas*, and *Nitrosospira*, which are known nitrifying bacteria (Chain et al., 2003; Starkenburg et al., 2006; Norton et al., 2008; Starkenburg et al., 2008; Lücker et al., 2013). Strain NJ1 is therefore unusual among nitrifying bacteria in that it lacks genes encoding bacterial flagella. Interestingly, it was reported that *Nitrospira* cells in strain NJ1 formed micro-colonies in the same manner as strain ND1 (Ushiki et al., 2013; Fujitani et al., 2014). Thus, it is likely that the potential for motility using flagella is not associated with micro-colony formation.

### CRISPR-Cas System of *Nitrospira* Genomes

The genome sequence of strain ND1 contained genes encoding the clustered regularly interspaced short palindromic repeat (CRISPR)-Cas (CRISPR-associated genes) systems, similar to those detected in *N. defluvii* and *N. moscoviensis* (Lücker et al., 2010; Koch et al., 2015) (**Figure 2**). By using CRISPRfinder (Grissa et al., 2007), it was confirmed that the genome sequence of strain ND1 contained CRISPR including 31 spacer sequences (crRNA) between the repeat units respectively (Supplementary Data Sheet S2). By contrast, the genome sequence of strain NJ1 contained no genes encoding the CRISPR-Cas system (**Figure 2**). The CRISPR-Cas system is a widely distributed prokaryotic immune system among bacteria and archaea. Likely, strain ND1 was equipped for resistance against phage using its own CRISPR-Cas system in the same manner as other *Nitrospira* isolates.

### Genomic Comparison between Strain ND1 and *Nitrospira defluvii*

Although several *Nitrospira* genomes were reported in the previous studies, the genome sequence of *Nitrospira* strains belonging to lineage I was only reconstructed from *N. defluvii*. Since the reported strain ND1 genome in this study was the second genome sequence of *Nitrospira* belonging to lineage I, homologous and non-homologous proteins between the two *Nitrospira* strains were identified by using the phyloprofile exploration tool of MicroScope (Vallenet et al., 2013). The genome sequence of strain ND1 contained 3,577 genes encoding homologous and 1,047 genes encoding non-homologous proteins with *N. defluvii*. The latter included the urease locus and the *nrfAH* gene mentioned above. In addition, genes encoding superoxide dismutase and catalase were also identified as non-homologous genes with *N. defluvii*. These two proteins are known as key enzymes for the defense against reactive oxygen species (ROS), and were also contained in the genome sequence of *N. moscoviensis* (Koch et al., 2015). By contrast, the genome sequence of *N. defluvii* contained 752 genes encoding non-homologous proteins with strain ND1, which included the *aoxAB* gene encoding large and small subunits of arsenite oxidase (AOX). It was reported that AOX functions in arsenite detoxification or enables *N. defluvii* to use arsenite as electron donor (Lücker et al., 2010).

Recently, genomics of *Nitrospira* strains belonging to lineage II revealed that *Nitrospira* bacteria possess hugely diverse functions such as utilization of formate or hydrogen, ammonia production from urea and cyanate, and ammonia oxidation (Koch et al., 2014, 2015; Daims et al., 2015; van Kessel et al., 2015). By contrast, due to insufficient genomic information, it has been unknown whether *Nitrospira* strains belonging to lineage I possess diverse functions. Owing to novel genomic information of strain ND1 revealed in this study, it is expected that *Nitrospira* lineage I possess genomic plasticity in the same manner as *Nitrospira* lineage II. Thus, further genomic information from *Nitrospira* strains belonging to lineage I and physiological characterization using available pure strains may help to illuminate still unknown functions of *Nitrospira* lineage I.

## Conclusion

In this study, the genome sequences of strains ND1 and NJ1 were reconstructed, and compared with those of other *Nitrospira* strains. Comparison of the genomes among *Nitrospira* lineages I and II revealed diversity of genes involved in urea degradation. Also, the comparative genomics illuminated the presence/absence of several functional genes/operons, such as motility, CRISPR-Cas system and QS system. In addition, although strains ND1 and NJ1 have been isolated from the same activated sludge, significant functional differences were predicted from their genomic information. These functional differences among the *Nitrospira* strains may be important factors in ecological niche differentiation among these bacteria in the activated sludge. Remarkably, we detected a gene encoding an AHL synthase involved in a QS system in the genome sequence of strain NJ1, and identified seven types of AHL within a range of C10–C14 from the supernatant of strain NJ1. Future transcriptional analyses using nitrifying pure cultures possessing QS systems may help to explain the mechanisms of transcriptional regulation and interspecies communication mediated by AHLs.

## Author Contributions

NU, HF, and ST designed the experiments. YSe performed and wrote the genome sequencing and assembly. NU performed the genome analysis. NU performed the gene expression experiments. NU, HF, and ST analyzed the data. YSh and TM identify acyl-homoserine lactones from *Nitrospira japonica* strain NJ1. NU drafted the manuscript. NU, HF, YSe, and ST read and edited the manuscript.

## Conflict of Interest Statement

The authors declare that the research was conducted in the absence of any commercial or financial relationships that could be construed as a potential conflict of interest.
