# Supplementary material for: Genomic Analysis of Two Phylogenetically Distinct Nitrospira Species Reveals Their Genomic Plasticity and Functional Diversity
Source: Front Microbiol. 2018 Jan 9;8:2637. doi: 10.3389/fmicb.2017.02637 (PMC5767232; doi:10.3389/fmicb.2017.02637)
Supplement: Supplementary file 1 [file Data_Sheet_1.docx]

*Supplementary Material*

Comparative genomics of *Nitrospira* strains: insight into their genomic plasticity and functional diversity

**Authors:** Norisuke Ushiki^1^, Hirotsugu Fujitani^1^, Yu Shimada^1^, Tomohiro Morohoshi^2^, Yuji Sekiguchi^3^ and Satoshi Tsuneda^1, *^

*** Correspondence:** Satoshi Tsuneda: stsuneda@waseda.jp

Materials and Methods

*Cultivation of* Nitrospira *strains*

Both strains were grown in 500 mL of batch culture in mineral medium composed of NaNO_2_ (49.3 mg L^-1^), KH_2_PO_4_ (38.2 mg L^-1^), MgSO_4_·7H_2_O (61.1 mg L^-1^), CaCl_2_·2H_2_O (10.0 mg L^-1^), FeSO_4_·7H_2_O (5.00 mg L^-1^), NaHCO_3_ (200 mg L^-1^), MnSO_4_·5H_2_O (54.2 µg L^-1^), H_3_BO_3_ (49.4 µg L^-1^), ZnSO_4_·7H_2_O (43.1 µg L^-1^), Na_2_Mo_4_O_4_ (27.6 µg L^-1^), and CuSO_4_·5H_2_O (25.0 µg L^-1^) (Ushiki et al., 2013; Fujitani et al., 2014). The pH was adjusted to 7.8 – 8.0 after autoclaving. The culture conditions were 29°C in the dark with shaking at 100 rpm. To investigate the ureolystic activity of strains ND1 and NJ1, each strain was incubated in 100 mL of batch culture containing mineral medium with urea (60.1 mg L^-1^) and NiCl_2_· 6H_2_O (60 µg L^-1^). The other culture conditions were the same as above. Every 2 days, aliquots of the incubations were sampled and filtered to remove cells through a 0.2-µm cellulose acetate membrane filter (Advantec, Tokyo, Japan).

*Chemical analysis*

Before chemical analysis, each sample was filtered through a membrane filter with a pore size of 0.22 μm (Advantec). The nitrite concentration of each filtered sample was determined photometrically using Griess reagent (Griess-Romijn, 1966). The total ammonium concentration of each filtered sample was determined photometrically using a salicylic acid assay (Kandeler and Gerber, 1988).

*Transcriptional analysis of genes in the strains ND1 and NJ1 genomes*

Each strain was incubated in nitrite medium with or without 10 mN L^-1^ NH_4_Cl under oxic conditions. The incubated samples were filtered through 0.2-µm cellulose acetate membrane filters (Advantec) for chemical analysis. The nitrite concentration of each filtered sample was determined photometrically with Griess reagent (Griess-Romijn, 1966). After the nitrite initially added had almost been consumed, total RNA was extracted from the incubated samples using an RNeasy Mini Kit (QIAGEN, Tokyo, Japan) according to the manufacturer’s instructions. After DNA in the extracted RNA samples was digested using gDNA Eraser (Takara Bio, Otsu, Japan), total RNA was reverse transcribed using the PrimeScript™ RT Reagent Kit (Takara Bio) according to the manufacturer’s instructions. Also, we checked no DNA left in any of the extracted RNA samples in by PCR. The reverse-transcribed single-stranded cDNA samples were purified using the Wizard SV Gel and PCR Clean up System (Promega, Tokyo, Japan). The concentrations of the purified cDNA samples were determined using a Qubit® ssDNA assay kit (Thermo Fisher Scientific, Yokohama, Japan) with a Qubit fluorometer (Thermo Fisher Scientific). The cDNA samples were diluted to 0.1 ng/µL with Tris-EDTA (TE) buffer and were used as a template for PCR. The following thermal profiles were used in each gene amplification using the respective primer sets detailed in Table S2: an initial denaturing step was conducted at 95°C for 2 min, followed by 40 cycles of denaturation at 95°C for 30 s, annealing at 50°C for 30 s, and elongation at 72°C for 30 s, and a final extension step was conducted at 72°C for 5 min. The PCR reaction mixtures (50 µL) contained 10× PCR buffer, 200 µM of each primer, 2.5 mM dNTPs, and Ex Taq DNA polymerase (Takara Bio).

*AHL bioassay*

An AHL bioassay using *C. violaceum* strain VIR07 was performed according to a previous study (Morohoshi et al., 2008). The *C. violaceum* strain VIR07 was inoculated into LB medium, and cultured overnight at 30°C. Then, 5 mL of the cultured strain VIR07 was mixed with 50 mL of LB medium containing 15 g L^-1^ agar, and poured as plates. Sterilized paper disks were placed onto the agar plate containing strain VIR07. Then, 10 μL of 10 μM C10-HSL or extracted samples the batch culture of strain NJ1 was applied to the paper disks, and incubated overnight at 30°C.

Results and Discussion

*Transcriptional analysis of genes involved in nitrogen metabolism*

To identify the nitrite reduction pathway in each strain, RNA was extracted from each isolate during incubation with or without ammonia, and the mRNA of genes involved in nitrogen metabolism was analyzed by reverse-transcription (RT)–PCR (Figure S1). RNA was extracted from each strain several days after incubation started, when added nitrite had been completely consumed (Figure S1A). Regardless of the presence or absence of ammonia in the medium, mRNA of the nitrite-oxidoreductase *β* subunit gene (*nxrB*) and the glutamine synthetase gene (*glnA*) of strain NJ1 were detected by RT-PCR (Figure S1B and C). By contrast, mRNA of the gene (NSJP_2412) encoding octaheme cytochrome *c* nitrite reductase (ONR) was detected in RNA extracted from strain NJ1 incubated in medium containing nitrite as the sole nitrogen source (Figure S1B and C), but not in medium with ammonia. This repression of NSJP_2412 gene expression is likely to be caused by ammonia utilization rather than nitrite reduction for nitrogen assimilation. Thus, strain NJ1 is most likely to use ONR encoded by NSJP_2412 for nitrite reduction. By contrast, mRNA of the *nirA* gene and the *nrfAH* gene of strain ND1 were not detected by RT-PCR (data not shown). However, nitrite reduction in strain ND1 was most likely to be catalyzed by NirA considering the spatial location of genes involved in nitrogen metabolism (Figure 3).

*Urea degradation by two* Nitrospira *strains*

The genome sequences of strains ND1 and NJ1 contained a gene cluster encoding urease and urease accessory protein (Figure 3), similar to *Nitrospira* *moscoviensis* and *Nitrospira lenta* (Koch et al., 2015). Urease usually consists of three subunits (alpha, beta, and gamma) encoded by a *ureABC* gene cluster, and the accessory protein consists of four subunits encoded by a *ureDEFG* gene cluster. Urease is a metalloenzyme that acquires nickel in its active site and is maturated by accessory protein (Mobley et al., 1995). It was reported that the lack of a partial accessory protein subunit significantly depresses ureolystic activity (Lee et al., 1992; Park et al., 1994). However, even if strain ND1 lacked the *ureE* gene, the ureolystic activity of strain ND1 was detected to a similar degree as that of strain NJ1 possessing a *ureE* gene (Figure S2). Similarly, *N. moscoviensis* possessed ureolystic activity despite lacking the *ureE* gene in their genomes. Recently, genomic analysis of *Nitrospira* suggested that hydrogenase metallochaperones might act as a substitute for UreE protein in *N.* *moscoviensis* (Koch et al., 2015). However, the strain ND1 genome contained no genes encoding metallochaperones.

Lee et al. (1992) reported that deletion of any of the *ureD*, *ureF*, or *ureG* genes led to the formation of an inactive urease, suggesting that UreD, UreF, and UreG are required for the complex assembly of the urease active site. By contrast, using equilibrium dialysis, it was confirmed that UreE bound to cellular nickel ions (Lee et al., 1993), and appeared to function as the nickel donor for urease maturation. Interestingly, comparing genomes among *Nitrospira* possessing urease loci, it was revealed that strain ND1 contained a gene (NSND_61682) encoding a putative high-affinity nickel/cobalt transporter, which was lacking in the strain NJ1 genome (Figure 2). Moreover, the genome of *N. moscoviensis* lacking the *ureE* gene contained not only this gene, but also the *ureH* gene encoding a nickel transporter. Thus, the urease of strain ND1 may be activated by enhancing the cellular nickel concentration by high-affinity nickel transport.

*Acyl-homoserine lactone* *(AHL) bioassays*

Before identification of AHLs in the batch culture of strain NJ1 using LC-MS/MS analysis, AHL bioassays were performed using *Chromobacterium violaceum* strain VIR07 as an AHL reporter cell (Morohoshi et al., 2008). Because it had been reported that the production of violacein in *C. violaceum* strain VIR07 was induced by adding long-chain AHLs (C10 – C16) (Morohoshi et al., 2008), C10-homoserine lactone (HSL) was used as a positive control. AHL bioassays with *C. violaceum* strain VIR07 confirmed that strain VIR07 produced violacein (violet color) after induction by C10-HSL (Figure S3A). Similarly, production of violacein in strain VIR07 was induced by the addition of the extracted sample the batch culture of strain NJ1 (Figure S3B). By contrast, as a negative control, strain VIR07 with no induction did not produced violacein (Figure S3C). The results of these bioassays therefore confirmed that the extracted sample the batch culture of strain NJ1 contained some long-chain AHLs (C10 – C16).

Reference

Burton, E. O., Read, H. W., Pellitteri. M. C., and Hickey, W. J. (2005). Identification of acyl-homoserine lactone signal molecules produced by *Nitrosomonas europaea* strain Schmidt. *Appl. Environ. Microbiol.* 71, 4906–4909.

Fujitani, H., Ushiki, N., Tsuneda, S., and Aoi, Y. (2014). Isolation of sublineage I *Nitrospira* by a novel cultivation strategy. *Environ. Microbiol.* 16, 3030-3040.

Gao, J., Ma, A., Zhuang, X., and Zhuang, G. (2014). An N-acyl homoserine lactone synthase in the ammonia-oxidizing bacterium *Nitrosospira multiformis*. *Appl. Environ. Microbiol.* 80, 951-958.

Griess-Romijn van Eck, E. (1966). (1966). *Physiological and Chemical Tests for Drinking Water. NEN 1056, IV-2*. Rijswijk: Nederlands Normalisatie Instituut.

Kandeler, E., and Gerber, H. (1988). Short-term assay of soil urease activity using colorimetric determination of ammonium. *Biol. Fertil. Soils* 6, 68–72.

Koch, H., Lücker, S., Albertsen, M., Kitzinger, K., Herbold, C., Spieck, E., et al. (2015). Expanded metabolic versatility of ubiquitous nitrite-oxidizing bacteria from the genus *Nitrospira*. *Proc. Natl. Acad. Sci. U. S. A.* 112, 11371-11376.

Lee, M. H., Mulrooney, S. B., Renner, M. J., Markowicz, Y., and Hausinger, R. P. (1992). *Klebsiella aerogenes* urease gene cluster: sequence of ureD and demonstration that four accessory genes (ureD, ureE, ureF, and ureG) are involved in nickel metallocenter biosynthesis. *J. Bacteriol.* 174, 4324-4330.

Lee, M. H., Pankratz, H. S., Wang, S., Scott, R. A., Finnegan, M. G., Johnson, M. K., et al. (1993). Purification and characterization of *Klebsiella aerogenes* UreE protein: a nickel-binding protein that functions in urease metallocenter assembly. *Protein Sci.* 2, 1042-1052.

Mellbye, B. L., Bottomley, P. J., and Sayavedra-Soto, L. A. (2015). Nitrite-oxidizing bacterium *Nitrobacter winogradskyi* produces n-acyl-homoserine lactone autoinducers. *Appl. Environ. Microbiol.* 81, 5917-5926.

Mellbye, B. L., Spieck, E., Bottomley, P. J., and Sayavedra-Soto, L. A. (2017). Acyl-Homoserine Lactone Production in Nitrifying Bacteria of the Genera *Nitrosospira*, *Nitrobacter*, and *Nitrospira* Identified via a Survey of Putative Quorum-Sensing Genes. *Appl. Environ. Microbiol.* 83:e01540-17.

Mobley, H. L., Island, M. D., and Hausinger, R. P. (1995). Molecular biology of microbial ureases. *Microbiol. Rev.* 59, 451-480.

Morohoshi, T., Kato, M., Fukamachi, K., Kato, N., and Ikeda, T. (2008). N-acylhomoserine lactone regulates violacein production in *Chromobacterium violaceum* type strain ATCC 12472. *FEMS Microbiol. Lett.* 279, 124-130.

Nasuno, E., Kimura, N., Fujita, M. J., Nakatsu, C. H., Kamagata, Y., and Hanada, S. (2012). Phylogenetically novel LuxI/LuxR-type quorum sensing systems isolated using a metagenomic approach. *Appl. Environ. Microbiol.* 78, 8067-8074.

Park, I. S., Carr, M. B., and Hausinger, R. P. (1994). In vitro activation of urease apoprotein and role of UreD as a chaperone required for nickel metallocenter assembly. *Proc. Natl. Acad. Sci. U. S. A.* 91, 3233-3237.

Pester, M., Maixner, F., Berry, D., Rattei, T., Koch, H., Lücker, S., et al. (2014). NxrB encoding the beta subunit of nitrite oxidoreductase as functional and phylogenetic marker for nitrite-oxidizing *Nitrospira*. *Environ. Microbiol.* 16, 3055-3071.

Shen, Q., Gao, J., Liu, J., Liu, S., Liu, Z., Wang, Y., et al. (2016). A new acyl-homoserine lactone molecule generated by *Nitrobacter winogradskyi*. *Sci. Rep.* 6:22903.

Ushiki, N., Fujitani, H., Aoi, Y., and Tsuneda, S. (2013). Isolation of *Nitrospira* belonging to sublineage II from a wastewater treatment plant. *Microbes Environ*. 28, 346-353.

Supplementary Figure legends

Figure S1

Nitrite consumption of strain NJ1 during incubation with nitrite or both nitrite and 10 mN L^-1^ NH_4_Cl (A). Circle plots show nitrite concentrations in the incubation with nitrite. Square plots show nitrite concentrations in the incubation with both nitrite and ammonia. A black arrow indicates the date RNA was extracted. RT-PCR data for the *nxrB*, NSJP_2412, and *glnA* genes (B and C). I, incubations with nitrite; II, incubations with both nitrite and ammonia; III, negative control without nucleic acids; M, size marker. The results of two biological replicates are shown for each incubation experiment.

Figure S2

Ureolytic activity of strain ND1 (A) or strain NJ1 (B) during incubation with urea and nitrite. Steady accumulation of ammonium was detected in the culture supernatant of both strains. Ammonium accumulation was not detected in the negative control experiments without the addition of biomass. The results of two biological replicates are shown for each incubation experiment.

Figure S3

Production of violacein in strain VIR07. AHL bioassay using *Chromobacterium violaceum* strain VIR07 with the addition of C10-HSL, as a positive control (A), with DMSO, as a negative control (B), and the extracted sample the batch culture of strain NJ1 (C).

Table S1 General genome features of strains ND1 and NJ1

|  | Strain ND1 | Strain NJ1 |
| --- | --- | --- |
| Genome size (bp) | 4,454,928 | 4,084,817 |
| Coverage | 92× | 160× |
| Number of scaffolds | 6 | 1 |
| Average G + C content (%) | 58.87 | 58.96 |
| Average CDS length (bp) | 878.35 | 901.26 |
| Average intergenic length (bp) | 121.07 | 112.08 |
| Protein coding density (%) | 89.55 | 90.33 |
| Number of genomic objects | 4680 | 4218 |
| (CDS, fCDS, rRNA, tRNA, miscRNA) |  |  |
| CDS | 4612 | 4150 |
| fCDS | 12 | 12 |
| rRNA | 3 | 3 |
| tRNA | 46 | 45 |
| misc_RNA | 7 | 8 |

CDS, coding sequences.

Table S2 Primer sets used for RT-PCR

| Primer name | Sequence (5ʹ–3ʹ) | Target gene | Length | Reference |
| --- | --- | --- | --- | --- |
| 169f | GCCATCAAGGAAACCTAT | *nxrB* | 470 bp | Pester et al., 2014 |
| 638r | AGCATCATGATGCGC |  |  |  |
| ONR_765f | AAACAAGGAAAAAATGAAGC | ONR  (NSJP_2412) | 270 bp | In this study |
| ONR_1034r | TGATCCATCGAAATAAAGAA |  |  |  |
| glnA_23f | AGTTTGCCAAAAAAAACAAG | *glnA* | 392 bp |  |
| glnA_414r | GATCGAAGATGAAGAACTC |  |  |  |

Table S3 Chromatographic and mass spectrometric data of acyl-HSLs identified by UHPLC-MS/MS in extracts from batch culture of *Nitrospira japonica* strain NJ1

| Acyl-HSL | Exact mass (Da) | [M+H]^+^(m/z)^a^ | Retention time (min) | Intensity^b^ |
| --- | --- | --- | --- | --- |
| C10-HSL | 255.4 | 256.2 | 5.68 | 2.998E+05 |
| 3-oxo-C10-HSL | 269.3 | 270.2 | 6.10 | 1.329E+05 |
| C12-HSL | 283.4 | 284.3 | 6.54 | 7.016E+06 |
| 3-oxo-C12-HSL | 297.4 | 298.2 | 6.99 | 9.034E+04 |
| 3-OH-C12-HSL | 299.4 | 300.1 | 5.42 | 1.770E+04 |
| C14-HSL | 311.5 | 312.2 | 7.47 | 4.011E+05 |
| 3-oxo-C14-HSL | 325.4 | 326.3 | 7.84 | 1.627E+04 |

a Experimental m/z values of protonated molecules

b Relative abundance of protonated molecules (unitless).

Table S4 List of identified AHLs from nitrifying bacteria

| Species (Reference) | | AHLs |
| --- | --- | --- |
| NOB | *Nitrospira japonica* strain NJ1  (in this study) | C10-HSL, 3-oxo-C10-HSL, C12-HSL, 3-oxo-C12-HSL, 3-OH-C12-HSL C14-HSL, 3-oxo-C14-HSL |
|  | Uncultured *Nitrospira* clone  (Nasuno et al., 2012) | C12-HSL |
|  | *Nitrospira moscoviensis*  (Mellbye et al., 2017) | C8-HSL |
|  | *Nitrobacter winogradskyi*  (Mellbye et al., 2015) | C10-HSL, monounsaturated C10-HSL |
|  | *Nitrobacter winogradskyi*  (Shen et al., 2016) | C7-HSL, C8-HSL, C9-HSL, C10-HSL, 7,8-trans-C10-HSL |
| AOB | *Nitrosomonas europaea*  (Burton et al., 2005) | C6-HSL, C8-HSL, C10-HSl |
|  | *Nitrosospira multiformis*  (Gao et al., 2014) | C14-HSL, 3-oxo-C14-HSL |

AOB, ammonia-oxidizing bacteria; NOB, nitrite-oxidizing bacteria.

Figure S1


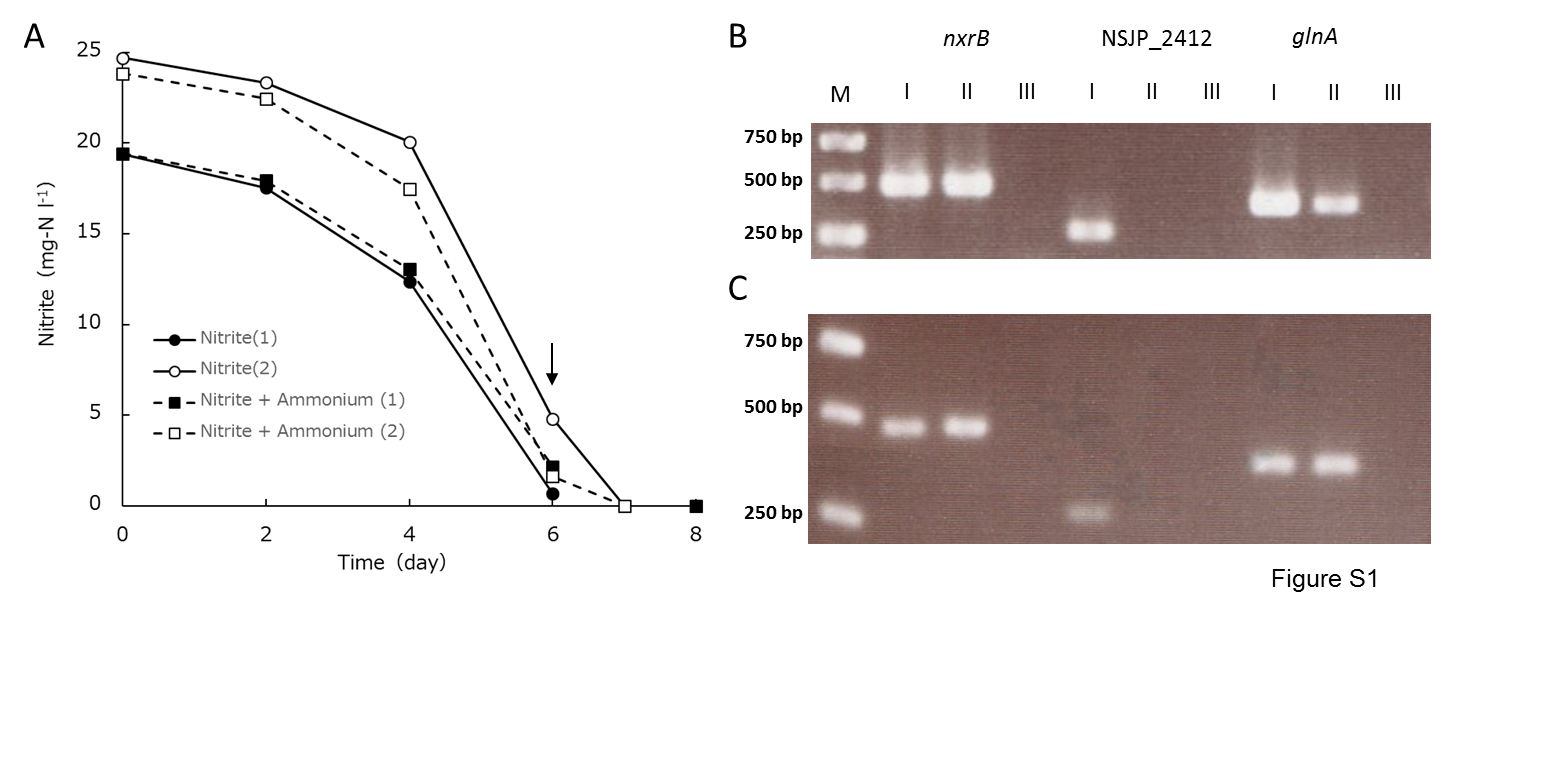


Figure S2


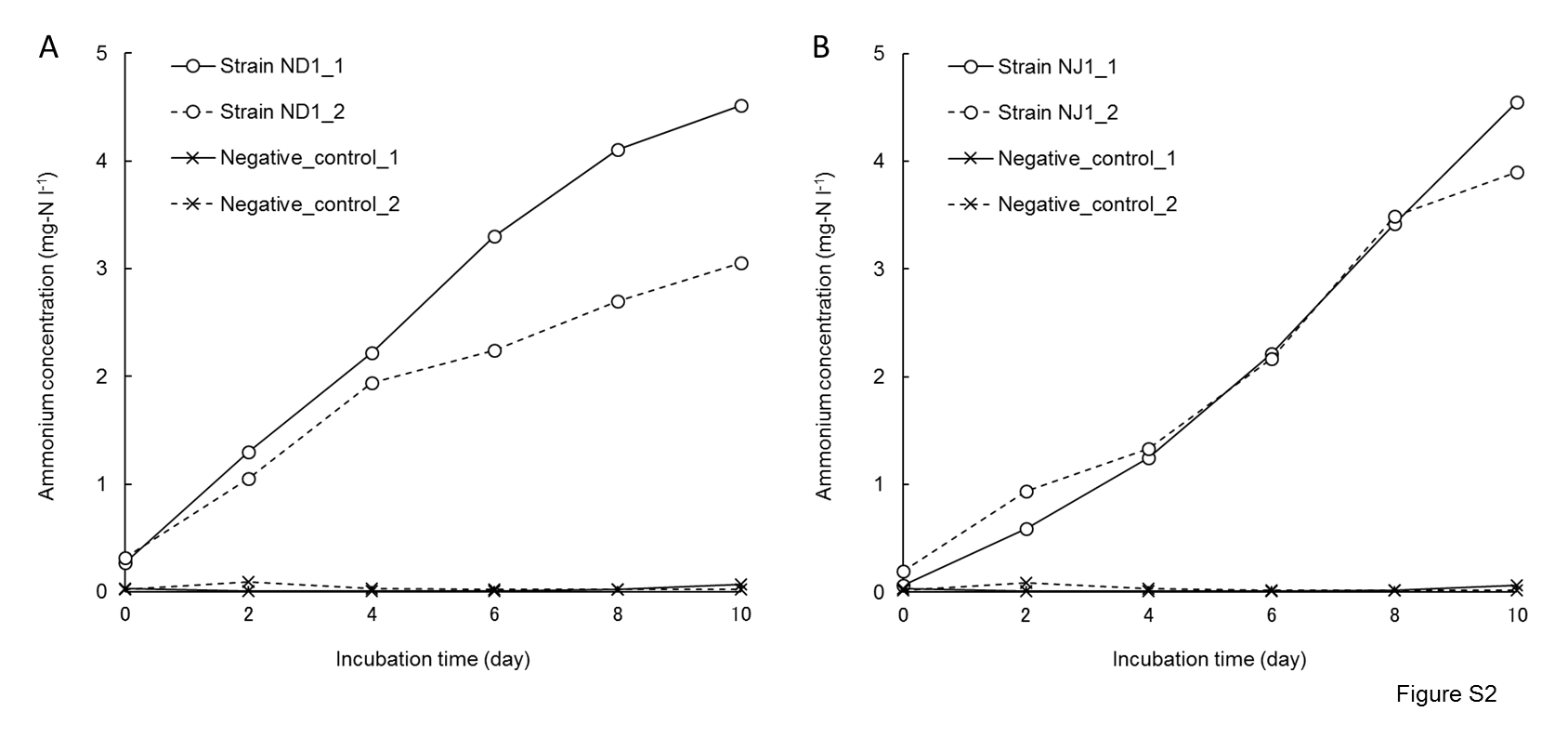


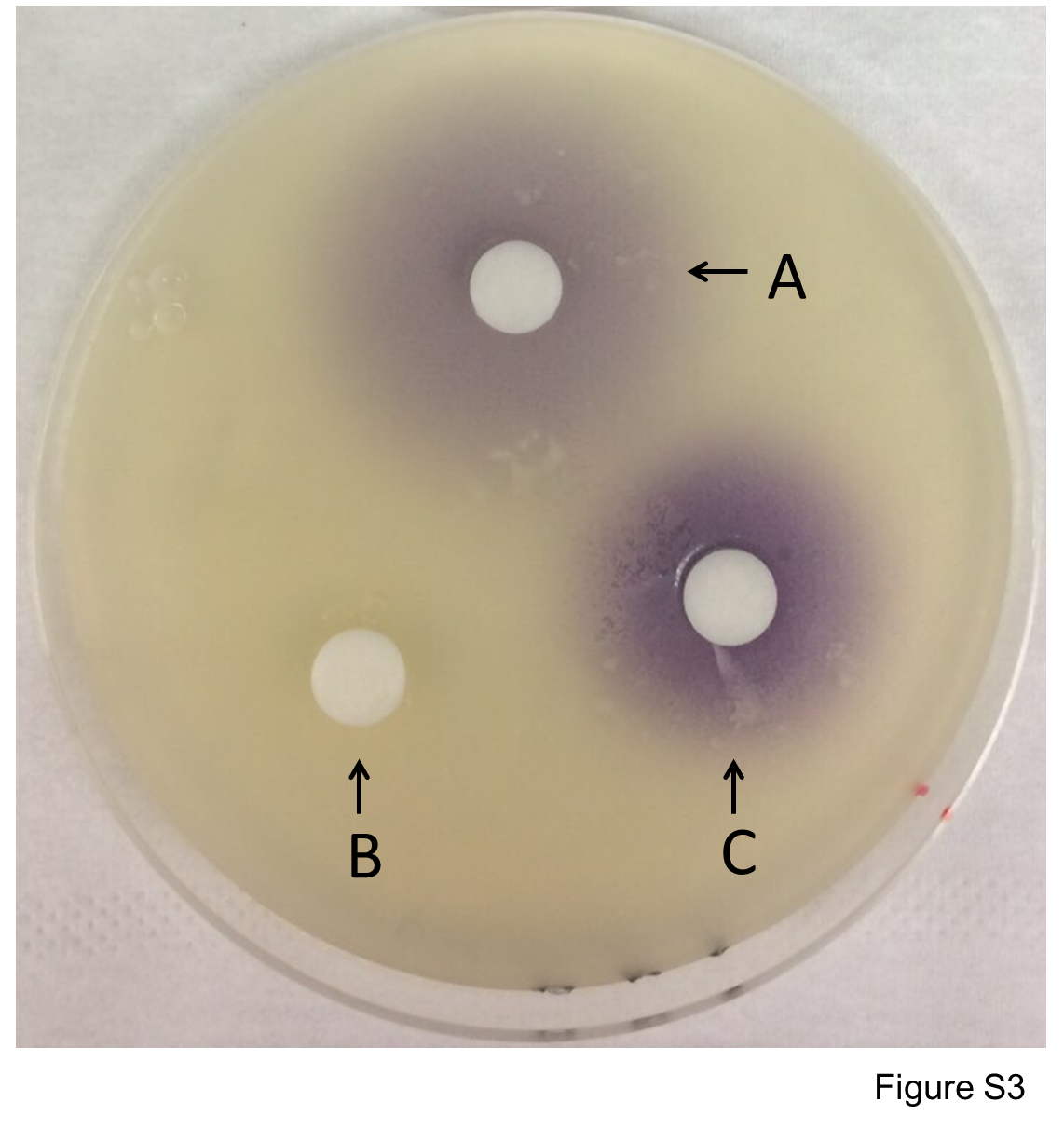
Figure S3
