# Supplementary figures and images for: Genomic Analysis of Two Phylogenetically Distinct Nitrospira Species Reveals Their Genomic Plasticity and Functional Diversity
Source: Front Microbiol. 2018 Jan 9;8:2637. doi: 10.3389/fmicb.2017.02637 (PMC5767232; doi:10.3389/fmicb.2017.02637)

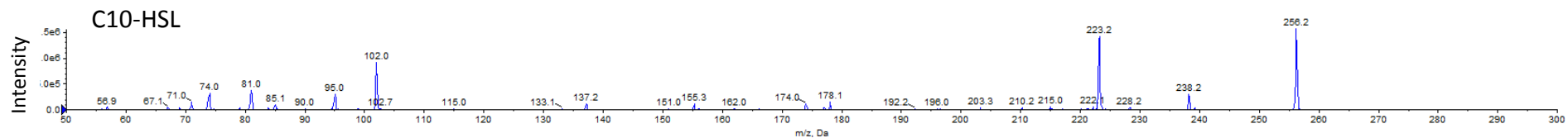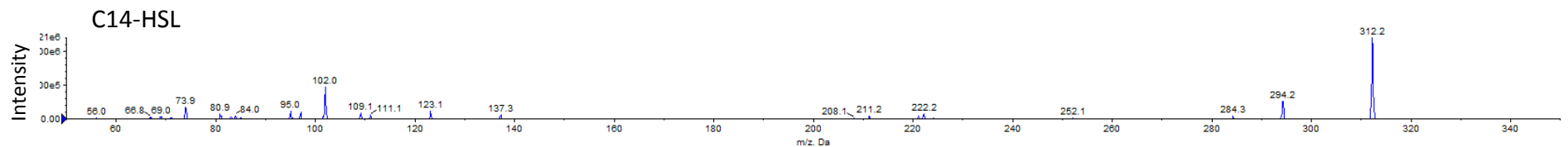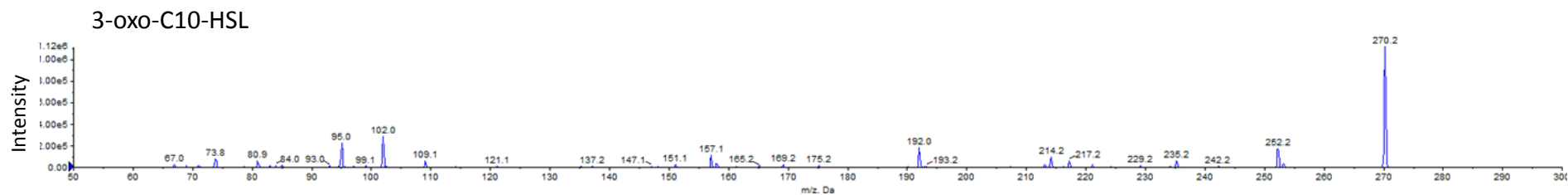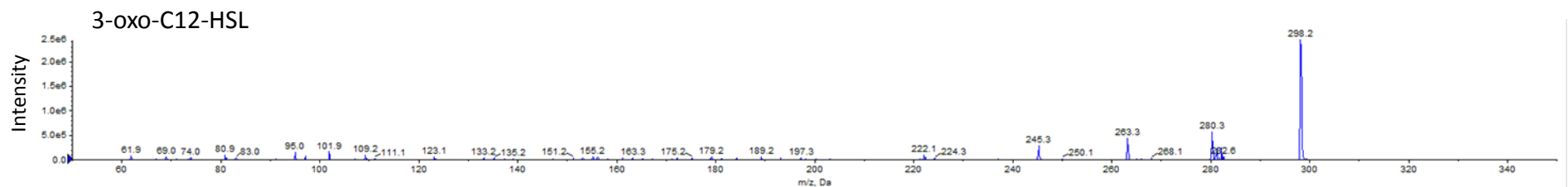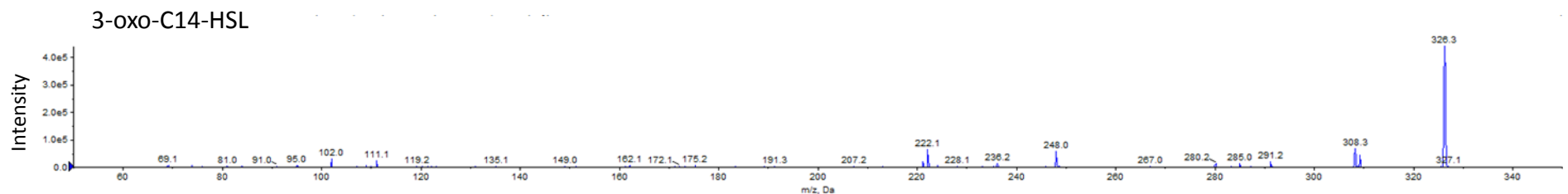

### C12-HSL

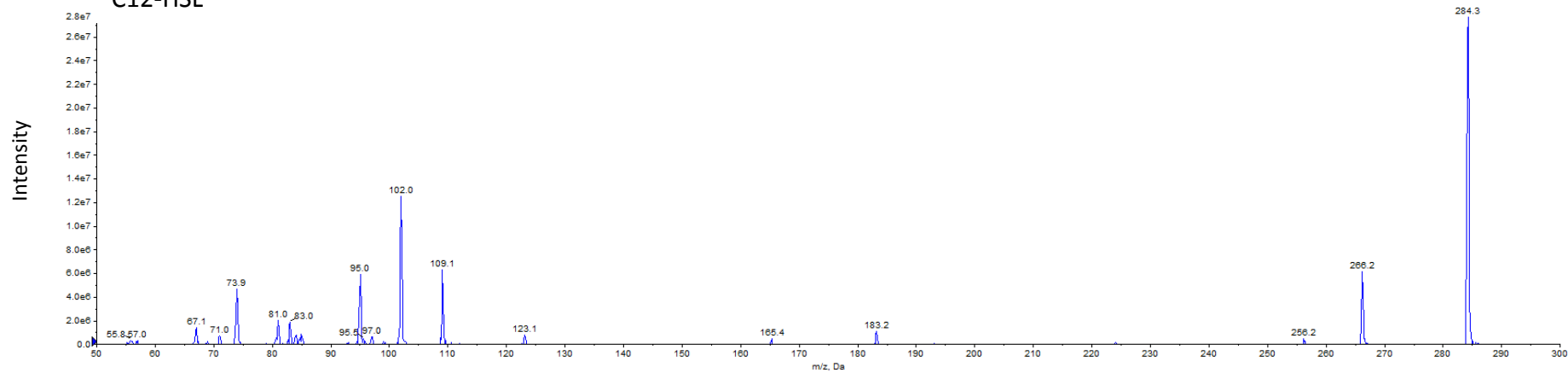

### 3-OH-C12-HSL

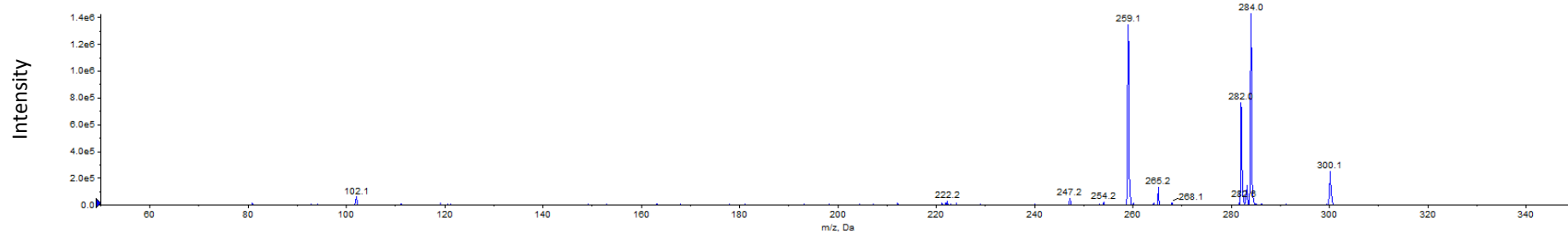

Supplement: Supplementary file 3 [file Data_Sheet_3.PDF]
